# Abcès primaire tuberculeux et à pyogène du psoas: une association exceptionnelle

**DOI:** 10.11604/pamj.2017.28.280.13796

**Published:** 2017-11-29

**Authors:** Christelle Mboyo Fataki, Zohour Kasmy, Sara Sahrourdi, Abdeljalil Raghani, Amal Rhars, Mohamed Frikh, Mariam Chadli, Abdelhay Lemnouar, Jilali Chaari, Mostafa Elouennass

**Affiliations:** 1 Laboratoire de Bactériologie, Hôpital Militaire d’Instruction Mohamed V, Rabat, Maroc; 2 Faculté de Médecine et de Pharmacie, Université Mohamed V, Rabat, Maroc; 3 Service de Médecine Interne, Hôpital Militaire d’Instruction Mohamed V, Rabat, Maroc

**Keywords:** Abcès du psoas, primaire, polymicrobien, tuberculeuse, pyogène, Psoas abscess, primary, polymicrobic, tuberculous, pyogenic

## Abstract

Les abcès du psoas représentent 5 à 10% des suppurations abdominales. Elles peuvent être primaires ou secondaires. Les abcès primaires polymicrobiens du psoas, notamment tuberculeux et à pyogène, n’ont jamais été rapportés. Nous en décrivons un cas chez un patient de 35 ans, sans antécédents pathologiques particuliers, admis pour la prise en charge des douleurs de la fosse lombaire droite associée à une fièvre à 40°C dont la symptomatologie remonte à 5 mois auparavant mais sans fièvre. La tomodensitométrie abdominale a montré un abcès des muscles psoas transverse et oblique externe droite étendu au retro-péritoine infiltrant la paroi thoraco-abdominale. L’analyse cytobactériologique du pus retrouve une culture riche et monomorphe d’*Escherichia coli* sauvage. La recherche du Complexe *Mycobaterium tuberculosis*effectuée systématiquement sur ce genre de prélèvement était positive après PCR tandis que l’examen direct après coloration de Ziehl Nelseen était négatif. La culture sur milieu solide Lowenstein-Jensen s’est positivée après un mois d’incubation. Le patient a bien évolué sous quadrithérapie antibacillaire et ceftriaxone. A travers ce cas, il en découle qu’une origine tuberculeuse doit être recherchée systématiquement en zone d’endémie devant tout abcès du psoas à évolution chronique, récidivant ou ne répondant pas aux antibiotiques.

## Abstract (en)

Psoas abscesses account for 5-10% of abdominal suppurations. They can be primary or secondary. Primary polymicrobic abscesses of the psoas muscle including, in particular, tuberculous abscesses and pyogenic abscesses, have never been reported in the literature. We report the case of a 35-year old patient, with no particular past medical history, admitted with pain in the right lumbar fossa associated with a fever of 40°C. Onset of symptoms had occurred 5 months before, but without fever. Abdominal CT scan showed an abscess of the right external transverse and oblique psoas muscles extended to the retroperitoneum and infiltrating the thoracoabdominal wall. Cytobacteriological examination of pus showed fast-growth monomorphic wild-type Escherichia coli strains. Systematically performed Real-time PCR test for the detection of Complex Mycobaterium tuberculosis was positive while direct examination after Ziehl-nelseen staining was negative. The culture on a solid Lowenstein Jensen medium was positive after one-month of incubation. The outcome of our patient was favorable under antibacillar quadritherapy and ceftriaxone. This study highlights that a tuberculous origin should be systematically suspected in patients living in endemic areas with chronic, recurrent psoas abscess not responding to antibiotics.

## Introduction

Les abcès du psoas sont peu fréquents et représentent moins de 10% des suppurations abdominales [1, 2]. Ils peuvent être primaires ou secondaire. L’abcès primaire du psoas est généralement dû à l’extension d’une infection intra ou rétro-péritonéale et est plus souvent monomicrobien [3]. Son diagnostic fait intervenir un faisceau d’arguments cliniques, radiologiques et biologiques. Les abcès primaires polymicrobiens du psoas, notamment tuberculeux et à pyogène, n’ont jamais été rapportés dans la littérature. Nous en décrivons un cas en insistant sur l’intérêt d’une investigation poussée dans la démarche diagnostique.

## Patient et observation

**Figure 1:**
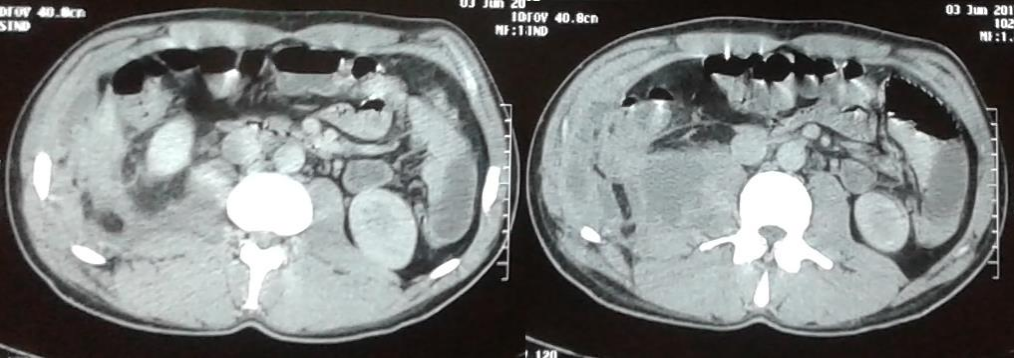
Tomodensitométrie abdominale révélant un abcès du psoas droit infiltrant la paroi thoraco-abdominale

Il s’agit d’un patient de 35 ans, sans antécédents pathologiques, admis pour la prise en charge des douleurs de la fosse lombaire droite associée à une fièvre à 40°C. Cette symptomatologie remonte à 5 mois auparavant mais évoluant sans fièvre. La tomodensitométrie (TDM) abdominale a objectivé des collections abcédées des muscles psoas transverse et oblique externe droite étendu au retro-péritoine infiltrant la paroi thoraco-abdominale (Figure 1). L’analyse cytobactériologique du pus collecté lors du drainage chirurgical retrouve une réaction cellulaire importante faite de polynucléaire avec une culture riche et monomorphe d’Escherichia coli sensible aux antibiotiques. La recherche du Complexe Mycobaterium tuberculosis effectuée systématiquement au laboratoire sur les pus du psoas a été réalisée. L’examen direct après coloration de Ziehl Nelseen était négatif. Cependant la recherche du génome du Complexe Mycobaterium tuberculosis par PCR en temps réel (GeneXpert^®^) sur le pus s’est avérée positive sans détection de la résistance à la rifampicine. La culture sur milieu solide Lowestein Jensen après un mois d’incubation était aussi positive. Le reste du bilan biologique a rapporté un syndrome inflammatoire avec une élévation de la CRP à 172,7 mg/L, une élévation de la vitesse de sédimentation (VS) à 80 mm à la première heure et une hyperleucocytose chiffrée à 18200 éléments/mm3 avec prédominance de polynucléaire neutrophile (16600/mm3). Les hémocultures réalisées lors des accès fébriles étaient négatives. Le bilan d’extension de la tuberculose était négatif. Le patient a été mis sous quadrithérapie antibacillaire (isoniazide 300 mg/j, rifampicine 600 mg/j, pyrazinamide 1500 mg/j et ethambutol 1200 mg/j) et ceftriaxone (2g/jour en intramusculaire). L’évolution était bonne avec diminution de la CRP et régression de la taille des abcès sur l’échographie.

## Discussion

Les abcès du psoas sont le plus souvent monomicrobiens et peuvent être primaires ou secondaires, en fonction de la présence ou de l'absence de maladie sous-jacente. Ils sont incriminés dans 5 à 10% des suppurations abdominales [1, 2]. La physiopathologie de l’abcès du psoas primaire n'est pas claire, mais la propagation lymphatique et hématogène d'un processus infectieux à partir d'une source occulte est évoqué. Ceci est associé dans la plupart des cas à des problèmes d’immunodépressions comme l'infection par le virus de l'immunodéficience humaine, les tumeurs malignes, l'insuffisance rénale, l'abus de drogues par voie intraveineuse, le diabète sucré et autres maladies chroniques ou traumatismes [4]. Mais aucune de ces pathologies n’a été retrouvée chez notre patient.

Les signes cliniques de l’abcès du psoas sont peu spécifiques. Le diagnostic est évoqué devant un état infectieux prolongé associé à des douleurs lombaires fébriles (abcès à pyogène) ou non (abcès froid) et au psoïtis. Comme chez notre patient, dans la majorité des cas, un syndrome inflammatoire biologique (élévation de CRP, VS, leucocytes) est présent mais reste généralement peu contributifs pour le diagnostic [4]. Le diagnostic positif de l’abcès est radiologique. La TDM a une sensibilité très élevée (environ 100%). Elle permet de mieux préciser l’extension de l’abcès, de déceler une lésion sous-jacente, de guider une ponction pour analyse microbiologique permettant le diagnostic étiologique et d’orienter la pose d’un drain à visée thérapeutique [5].

L’examen cytobactériologique du pus collecté permet d’isoler le germe incriminé. Il est effectué de préférence avant de débuter une antibiothérapie ou après une fenêtre d’arrêt. Le staphylocoque est le germe le plus incriminé dans les abcès primaires du psoas (90% des cas), suivi des streptocoques (5%) et d'*Escherichia coli*(3%) [6-8]. D'autres germes sont rarement impliqués dans les abcès primaires du psoas tels que *Brucella sp* et le Complexe *Mycobactérium tuberculosis* [9]. Dans la littérature, les abcès primaires rencontrés sont monomicrobiens. Chez notre patient, nous avons isolés à la fois un pyogène (*Escherichia coli*) et une mycobactérie du Complexe *Mycobactérium tuberculosis*. Etant donné l’absence de fièvre au début de la symptomatologie et l’évolution chronique de l’abcès, l’hypothèse la plus probable est une origine primitive tuberculeuse suivie d’une surinfection par un *Escherichia coli* endogène lors de l’infiltration de la paroi thoraco-abdominale comme le rapporte la TDM. L'amélioration des techniques de diagnostic biologique a facilité ce diagnostic. En effet, les technologies de biologie moléculaire par amplification génique (PCR) en temps réel que nous avons réalisées permettent de détecter rapidement la présence d'un nombre très faible de séquences génomiques et de rendre rapidement les résultats (en 2 heures pour GeneXpert^®^). Il est rapporté que la sensibilité de la PCR est d’environ 70% lorsque l’examen direct est négatif comme chez notre patient [10]. La PCR a donc permis une meilleure prise en charge du patient grâce à la mise en route précoce du traitement antituberculeux.

Classiquement, la recherche du complexe *Mycobactérium tuberculosis* se fait par examen direct après coloration de Ziehl Nielsen suivi par la culture sur milieux solides (Löwenstein-Jensen) ou liquides. La culture est la technique de référence pour le diagnostic de certitude mais elle est longue (1 à 2 mois). L’examen direct est rapide et permet de mettre en route le traitement. Cependant, il est peu sensible, n’étant positif que lorsque la concentration bacillaire est au moins égale à 104/mL. D’où, la recherche négative lorsque les prélèvements sont paucibacillaires comme couramment rencontré dans la tuberculose extra-pulmonaire. Cela explique sa négativité sur notre prélèvement. Actuellement, le traitement des abcès primitifs repose sur une antibiothérapie associée au drainage percutané. Notre patient a bien répondu aux traitements antituberculeux associé à la ceftriaxone. Dans la plupart des cas rapportés dans la littérature, l’abcès du psoas primitif semble avoir un meilleur pronostic que ceux qui sont secondaires à d'autres maladies [2].

## Conclusion

L’originalité de notre observation tient dans l’incrimination à la fois du Complexe *Mycobacterium tuberculosis* et *d’Escherichia coli* dans la genèse de l’abcès primaire du psoas. Devant tout abcès à évolution chronique, récidivant ou ne répondant pas à une antibiothérapie, une origine tuberculeuse doit être recherchée systématiquement surtout en zone d’endémie tuberculeuse. L’apport de la biologie moléculaire est considérable pour la prise en charge des tels abcès.

## Conflits d’intérêts

Les auteurs ne déclarent aucun conflit d'intérêts.
